# A contribution to the genus *Steccherinum* (Steccherinaceae, Polyporales): Introducing two new species and two new combinations of the genus

**DOI:** 10.3389/fmicb.2023.1166267

**Published:** 2023-03-22

**Authors:** Zhan-Bo Liu, Meng Zhou, Qiu-Yue Zhang, Jing Si

**Affiliations:** Institute of Microbiology, School of Ecology and Nature Conservation, Beijing Forestry University, Beijing, China

**Keywords:** diversity, macrofungi, phylogenetic analysis, wood-rotting fungi, fungal resources

## Abstract

Two new wood-inhabiting fungi from China, *Steccherinum juniperi* and *S. incrustans*, in the family Steccherinaceae are described and illustrated based on morphological and molecular analyses. The species *S. juniperi* was found growing on the rotten wood of *Juniperus* in Qinghai Province, China, while *S. incrustans* was collected on rotten angiosperm wood in Yunnan Province, China. The characteristics of *S. juniperi* include annual, resupinate basidiomata with a buff yellow fresh pore surface that becomes apricot orange when bruised, angular pores of 3–6 per mm, subicular generative hyphae sometimes covered with crystals, the presence of encrusted skeletocystidia in tube trama only, fusiform to slim clavate cystidioles, and ellipsoid basidiospores measuring as 3–4 × 2–3 μm. The characteristics of *S. incrustans* include annual, resupinate basidiomata with a buff yellow or pinkish buff to clay buff dried pore surface, angular pores (8–10 per mm), generative hyphae in trama frequently covered with crystals, the presence of encrusted skeletocystidia in tube trama and hymenium, and ellipsoid basidiospores (3.5–4.5 × 2.5–3.5 μm). Phylogenetic analysis based on a combined 2-locus dataset [ITS1-5.8S-ITS2 (ITS) + nuclear large subunit RNA (nLSU)] shows that the two species are members of *Steccherinum*, and they are compared with morphologically similar and related species of this genus, respectively. In addition, two new combinations from *Junghuhnia*, transferred to *Steccherinum* as *S. austrosinense* and *S. nandinae*, are proposed based on examination of their type materials and phylogenetic analysis.

## 1. Introduction

*Steccherinum* Gray was established by Gray (1821), with *S. ochraceum* (Pers. ex J.F. Gmel.) Gray selected as its type. It is the largest genus in the Steccherinaceae (Polyporales) and has a worldwide distribution, with ~76 species accepted by Index Fungorum (http://www.indexfungorum.org/; accessed on 1 January 2023) and MycoBank (https://www.mycobank.org; accessed on 1 January 2023). Dai (2011) summarized corticioid and hydnoid fungi in China and 12 species of *Steccherinum* were mentioned. An identification key to 15 species of *Steccherinum* recorded from China was provided (Wan and Yuan, 2013).

*Steccherinum* is characterized by the resupinate to effuse-reflexed basidiomata with poroid or odontioid to hydnoid hymenophore, a monomitic or dimitic hyphal structure with thick-walled skeletal hyphae; most species have clamped generative hyphae, encrusted or smooth skeletocystidia, and smooth, thin-walled, ellipsoid basidiospores (Maas Geesteranus, 1974; Eriksson et al., 1984; Miettinen et al., 2012).

Miettinen et al. (2012) carried out a multigene phylogenetic analysis (ITS + nLSU + mtSSU + atp6 + tef1) for Steccherinaceae and proposed the monophyletic *Steccherinum* clade (Figure 4 in Miettinen et al., 2012). Liu and Dai (2021) thought that the limit of the genus *Steccherinum* in Miettinen et al. (2012) is reasonable, described *S. fragile* Z.B. Liu & Y.C. Dai, and proposed *S. subcollabens* (F. Wu et al.) Z.B. Liu & Y.C. Dai within the *Steccherinum* clade in their phylogenetic analysis of ITS + nLSU. Subsequently, Wu et al. (2021) described *S. puerense* Y.X. Wu et al. and *S. rubigimaculatum* Y.X. Wu et al. Dong et al. (2022) described *S. hirsutum* Y.X. Wu & C.L. Zhao and *S. yunnanense* Y.X. Wu & C.L. Zhao based on their phylogenetic analyses.

During investigations on the diversity of wood-rotting fungi from China, three resupinate polypore specimens were collected from Yunnan Province and Qinghai Province. Their morphology corresponded to the concept of *Steccherinum*. To confirm their affinity, phylogenetic analysis based on the ITS and nLSU rDNA sequences was carried out. Both morphological characteristics and molecular evidence demonstrated that these three specimens represent two new species of *Steccherinum*, which we describe in the present study. In addition, we studied the type specimens of *Junghuhnia austrosinensis* F. Wu et al. and *J. nandinae* F. Wu et al. They were transferred to *Steccherinum* based on morphological and phylogenetic analyses.

## 2. Materials and methods

### 2.1. Morphological studies

Macro-morphological descriptions were based on dry herbarium specimens and field notes. Microscopic measurements and drawings were prepared from slide preparations of dried tissues stained with Cotton Blue and Melzer's reagent as described by Dai (2010). Pores were measured by subjectively choosing as straight a line of pores as possible and measuring how many per mm. The following abbreviations are used in the description: CB = Cotton Blue; CB+ = cyanophilous in Cotton Blue; CB– = acyanophilous in Cotton Blue; IKI = Melzer's reagent; IKI– = neither amyloid nor dextrinoid in Melzer's reagent; KOH = 5% potassium hydroxide; n (a/b) = number of spores (a) measured from given number of specimens (b); L = spore length (arithmetic average of all the spores); W = spore width (arithmetic average of all the spores); and Q = variation in the L/W ratios between the specimens studied. When the variation in spore size is shown, 5% of the measurements were excluded from each end of the range, and these values are shown in parentheses. Special color terms follow Petersen (1996), and then, herbarium abbreviations follow Thiers (2018). Voucher specimens from the study were deposited in the herbarium of the Institute of Microbiology, Beijing Forestry University (BJFC).

### 2.2. DNA extraction, PCR amplification, and sequencing

Total genomic DNA was extracted from dried specimens using a CTAB Rapid Plant Genome Extraction Kit (Aidlab Biotechnologies Company, Ltd., Beijing, China) according to the manufacturer's instructions with some modifications (Li et al., 2014). The ITS regions were amplified with primers ITS4 and ITS5 (White et al., 1990). The nLSU regions were amplified with primers LR0R and LR7 (Vilgalys and Hester, 1990).

The polymerase chain reaction (PCR) procedure for the ITS was as follows: initial denaturation at 95°C for 3 min, followed by 35 cycles at 94°C for 40 s, 54°C for 45 s, and 72°C for 1 min, and a final extension of 72°C for 10 min. The PCR procedure for the nLSU was as follows: initial denaturation at 94°C for 1 min, followed by 35 cycles at 94°C for 30 s, 48°C for 1 min, and 72°C for 1.5 min, and a final extension of 72°C for 10 min (Zhao et al., 2015). Aliquots of PCR products were examined on 2% agarose gels stained with GelStar Nucleic Acid Gel Stain (Lonza Rockland, Inc., Rockland, YN, USA) and examined under UV light. The sequencing of the PCR products was conducted by the Beijing Genomics Institute, Beijing, China, with the same primers used in the PCR reactions. Species were identified by sequence comparison with accessions in the NCBI databases using the BLAST program.

### 2.3. Phylogenetic analyses

Phylogenetic trees were constructed using ITS + nLSU rDNA sequences, and phylogenetic analyses were performed with the maximum likelihood (ML), maximum parsimony (MP), and Bayesian inference (BI) methods. Sequences of the species and strains were primarily adopted from ITS-based and 28S-based tree topology as described by Liu and Dai (2021). New sequences generated in this study, along with reference sequences retrieved from GenBank (https://www.ncbi.nlm.nih.gov/genbank/; Table 1), were aligned by MAFFT 7 (Katoh et al., 2019; http://mafft.cbrc.jp/alignment/server/) using the “G-INS-i” strategy and manually adjusted in BioEdit 7.2.5 (Hall, 1999). Unreliably aligned sections were removed before the analyses, and efforts were made to manually inspect and improve the alignment. The data matrix was edited in Mesquite 3.70 (https://www.mesquiteproject.org/; Maddison and Maddison, 2021). The sequence alignment was deposited at TreeBase (Submission ID: 30018). According to Miettinen et al. (2012), *Junghuhnia crustacea* (Jungh.) Ryvarden also belongs to the family Steccherinaceae and is not close to the *Steccherinum* clade, thus sequences of *Junghuhnia crustacea* obtained from GenBank were used as out-groups to root the trees in the ITS + nLSU analysis.

**Table 1:** List of species, specimens, and GenBank accession numbers of the sequences used in this study.

**Species**	**Sample no**.	**GenBank no**.		**References**
**ITS**	**nLSU**
*Junghuhnia crustacea*	Miettinen 13852, 1	JN710554	JN710554	Miettinen et al., 2012
*J*. *crustacea*	Miettinen 2954, 1	JN710553	JN710553	Miettinen et al., 2012
*Steccherinum amapaense*	M245	KY977406	KY977405	Hyde et al., 2017
*S. amapaense*	AS888	–	KY980666	Hyde et al., 2017
***S. austrosinense***	**Dai 17540**	**MN871755**	**MN877768**	**Du et al.**, **2020**
***S. austrosinense***	**Dai 17679**	**MN871756**	**MN877769**	**Du et al.**, **2020**
*S. autumnale*	Spirin 2957	JN710549	JN710549	Miettinen et al., 2012
*S. bourdotii*	HHB9743sp	KY948818	–	Justo et al., 2017
*S. collabens*	KHL 11848	JN710552	JN710552	Miettinen et al., 2012
*S. fimbriatellum*	Miettinen 2091	JN710555	JN710555	Miettinen et al., 2012
*S. formosanum*	TFRI 652	EU232184	EU232268	Westphalen et al., 2019
*S. fragile*	Dai 20479	MW364628	MW364626	Liu and Dai, 2021
*S. fragile*	Dai 19972	MW364629	MW364627	Liu and Dai, 2021
*S. hirsutum*	CLZhao 4222	MW290040	MW290054	Dong et al., 2022
***S. incrustans***	**Miettinen 10301**	**JN710550**	**JN710550**	**Miettinen et al.**, **2012**
***S. incrustans***	**Dai 19442**	**ON182084** ^*^	**ON182087** ^*^	**Present study**
***S. juniperi***	**Dai 23930**	**OP956076** ^*^	**–**	**Present study**
***S. juniperi***	**Dai 23931**	**OP956077** ^*^	**OP956031** ^*^	**Present study**
*S. lacerum*	Niemela 8246	JN710557	JN710557	Miettinen et al., 2012
*S. larssonii*	MCW 593/17	MT849306	MT849306	Westphalen et al., 2021
*S. larssonii*	MCW 594/17	MT849307	MT849307	Westphalen et al., 2021
*S. meridionale*	CBS 125887	MH864086	MH875544	Vu et al., 2019
*S. meridionale*	MR 284	KY174992	KY174992	Westphalen et al., 2018
***S. nandinae***	**Dai 21107**	**MN833677**	**MN833679**	**Du et al.**, **2020**
***S. nandinae***	**Dai 21108**	**MN833678**	**MN833680**	**Du et al.**, **2020**
*S. neonitidum*	MCW 371/12	KY174990	KY174990	Westphalen et al., 2018
*S. nitidum*	MT 33/12	KY174989	KY174989	Westphalen et al., 2018
*S. nitidum*	KHL 11903	JN710560	JN710560	Miettinen et al., 2012
*S. ochraceum*	KHL 11902	JN710590	JN710590	Miettinen et al., 2012
*S. ochraceum*	2060	JN710589	JN710589	Miettinen et al., 2012
*S. polycystidiferum*	RP 140	KY174996	KY174996	Westphalen et al., 2018
*S. polycystidiferum*	MCW 419/12	KY174995	KY174995	Westphalen et al., 2018
*S. pseudozilingianum*	Kulju 1004	JN710561	JN710561	Miettinen et al., 2012
*S. puerense*	CLZhao 3122	MW682341	–	Wu et al., 2021
*S. puerense*	CLZhao 3644	MW682342	MW682338	Wu et al., 2021
*S. rubigimaculatum*	CLZhao 4069	MW682343	MW682339	Wu et al., 2021
*S. rubigimaculatum*	CLZhao 10638	MW682344	MW682340	Wu et al., 2021
*S. subcollabens*	Dai 19345	MN871759	MN877772	Du et al., 2020
*S. subcollabens*	Dai 19344	MN871758	MN877772	Du et al., 2020
*S. tenue*	KHL 12316	JN710598	JN710598	Miettinen et al., 2012
*S. tenue*	FP102082sp	KY948817	–	Justo et al., 2017
*S. tenuispinum*	Miettinen 8065, 2	JN710599	JN710599	Miettinen et al., 2012
*S. tenuispinum*	Spirin 2116	JN710600	JN710600	Miettinen et al., 2012
*S. undigerum*	MCW 426/13	KY174986	KY174986	Westphalen et al., 2018
*S. undigerum*	MCW 496/14	KY174988	KY174988	Westphalen et al., 2018
*S. yunnanense*	CLZhao 1445	MW290042	MW290056	Dong et al., 2022
*S. yunnanense*	CLZhao 2822	MW290043	MW290057	Dong et al., 2022

* Newly generated sequences for this study. New taxa and new combinations are in bold.

Maximum likelihood analysis was conducted using RAxML-HPC 8.2.3 (Stamatakis, 2014) and RAxML-HPC through the CIPRES Science Gateway 3.3 (Miller et al., 2010; http://www.phylo.org). Statistical support values were obtained using non-parametric bootstrapping with 1,000 replicates. The BI analysis was performed with MrBayes 3.2.7a (Ronquist et al., 2012). Four Markov chains were run for two runs from random starting trees for 1 million generations until the split deviation frequency value <0.01, and the trees were sampled at every 1,000 generations. The first 25% of the sampled trees were discarded as burn-in, and the remaining ones were used to reconstruct a majority rule consensus tree and calculate the Bayesian posterior probabilities (BPP) of the clades.

Maximum parsimony analysis was applied to the ITS + nLSU dataset sequences. The approaches to phylogenetic analysis were conducted as described by Liu et al. (2022), and the tree was constructed using PAUP^*^ 4.0β10 (Swofford, 2002). All the characters were equally weighted, and gaps were treated as missing data. Trees were inferred using the heuristic search option with tree bisection and reconnection branch swapping, and 1,000 random sequence addition maxtrees were set to 5,000. Branches of zero length were collapsed, and all the parsimonious trees were saved. Clade robustness was assessed using a bootstrap analysis with 1,000 replicates (Felsenstein, 1985). Descriptive tree statistics, including the consistency index (CI), homoplasy index (HI), rescaled consistency index (RC), retention index (RI), and tree length (TL), were calculated for each maximum parsimonious tree generated.

A total of 24 models of evolution were scored using PAUP^*^ 4.0β10 (Swofford, 2002). Optimal substitution models for the combined dataset were then determined using the Akaike information criterion implemented in MrModeltest 2.3 (Posada and Crandall, 1998; Nylander, 2004). The model GTR + I + G was selected in the ML and BI analyses.

Branches are labeled with ML bootstrap ≥ 70%, MP bootstrap ≥ 50%, and BPP ≥ 0.95, respectively. FigTree 1.4.4 (Rambaut, 2018) was used to visualize the resulting tree.

## 3. Results

### 3.1. Phylogenetic results

The combined ITS + nLSU dataset included sequences from 47 specimens representing 27 species (Table 1). The dataset had an aligned length of 2,044 characters, of which 1,544 were constant, 96 were variable but parsimony-uninformative, and 404 were parsimony-informative. MP analysis yielded an equally parsimonious tree (CI = 0.524, HI = 0.476, RC = 0.403, RI = 0.768, TL = 1,288). ML analysis resulted in the best tree, and Bayesian and MP analyses resulted in a similar topology to the ML analysis, with an average standard deviation of split frequencies of 0.007184 (BI). Hence, the ML tree is shown combined with the support values from the MP and BI analyses.

**Figure 1:**
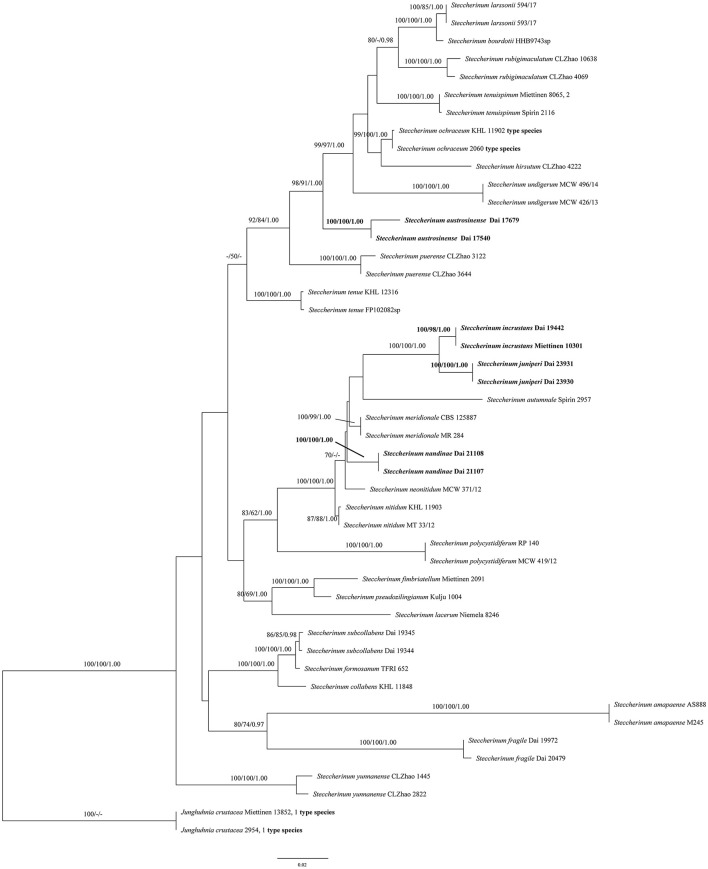
Phylogeny of *Steccherinum* generated by maximum likelihood (ML) analysis based on combined ITS and nLSU rDNA sequences. Branches are labeled with ML bootstrap >70%, maximum parsimony bootstrap >50%, and Bayesian posterior probabilities >0.95, respectively. New species and new combinations are in bold.

The phylogeny (Figure 1) inferred from the ITS and nLSU sequences demonstrated that the new species (*Steccherinum juniperi* and *S. incrustans*) and new combinations (*S. austrosinense* and *S. nandinae*) clustered in *Steccherinum* clade, and thus, they are described and proposed herein.

### 3.2. Taxonomy

***Steccherinum incrustans*** Z.B. Liu, Y.C. Dai & Jing Si, sp. nov.

MycoBank: MB847683.

Figures 2, 3.

**Figure 2:**
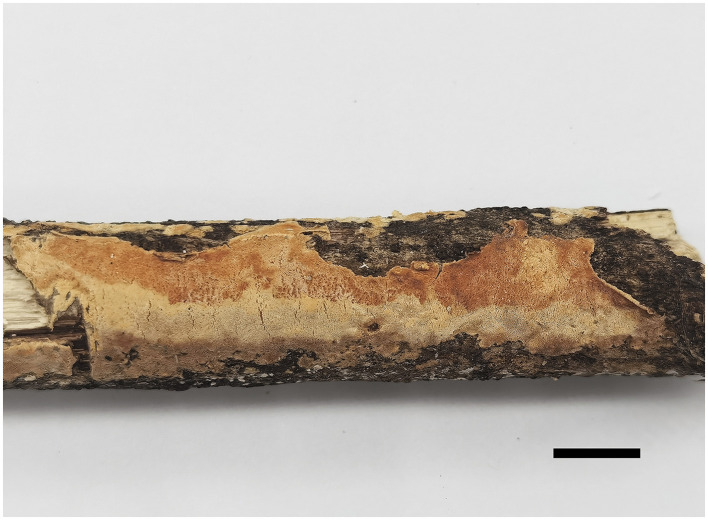
Basidiomata of *Steccherinum incrustans* (Holotype, Dai 19442). (Scale bar = 1.0 cm). Photographed by Meng Zhou.

**Figure 3:**
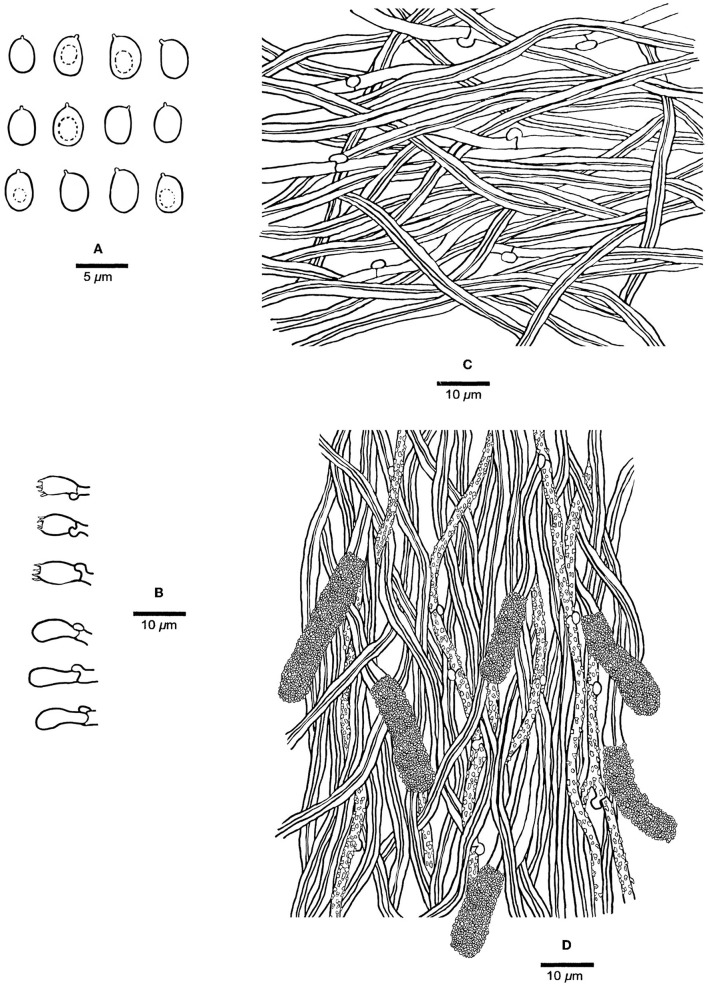
Microscopic structures of *Steccherinum incrustans* (Holotype, Dai 19442). **(A)** Basidiospores. **(B)** Basidia and basidioles. **(C)** Hyphae from subiculum. **(D)** Skeletocystidia and hyphae from trama. Drawn by Meng Zhou.

*Holotype*—China. Yunnan Province, Jinghong, Xishuangbanna Tropical Botanical Garden, on rotten angiosperm wood, 16.XII.2018, Dai 19442 (BJFC027910).

*Etymology—Incrustans* (Lat.): referring to the species having encrusted generative hyphae in trama.

*Fruiting body*—Basidiomata annual, resupinate, difficult to separate from the substrate, soft corky when fresh, hard corky when dry, up to 11 cm long, 2 cm wide, and ~1.5 mm thick at the center; pore surface buff yellow or pinkish buff to clay buff upon drying; sterile margin indistinct; pores angular, 8–10 per mm; dissepiments thin, entire; subiculum very thin to almost absent, paler than tubes, nearly 0.2 mm thick; tubes concolorous with poroid surface, up to 1.3 mm long.

*Hyphal structure*—Hyphal system dimitic; generative hyphae with clamp connections; skeletal hyphae dominant, CB+, IKI–; tissues unchanged in KOH.

*Subiculum*—Generative hyphae hyaline, thin-walled, unbranched, 2–3 μm in diam; skeletal hyphae dominant, hyaline, thick-walled with a narrow lumen, unbranched, flexuous, interwoven, 2.5–3.5 μm in diam.

*Tubes*—Generative hyphae hyaline, thin-walled, rarely branched, frequently, and strongly encrusted with crystals, 1–2.5 μm in diam; skeletal hyphae dominant, hyaline, thick-walled with a narrow lumen, unbranched, flexuous, interwoven, 2–3 μm in diam. Skeletocystidia present in the hymenium and trama, abundant, clavate to cylindrical, thick-walled with a narrow lumen, originated from tramal skeletal hyphae, then projecting from hymenium, strongly encrusted in the obtuse apex, 15–35 × 5–10 μm (encrusted part); cystidioles absent; basidia barrel-shaped, hyaline, with a basal clamp connection and four sterigmata, 9–13 × 4–5.5 μm; basidioles dominant, clavate similar with basidia in length.

*Spores*—Basidiospores ellipsoid with an apiculus, hyaline, thin-walled, smooth, some with a medium guttule, IKI–, CB–, (3–)3.5–4.5(−4.7) × (2.2–)2.5–3.5(−3.8) μm, L = 3.98 μm, W = 2.90 μm, Q = 1.37 (n = 60/1).

***Steccherinum juniperi*** Z.B. Liu, Y.C. Dai & Jing Si, sp. nov.

MycoBank: MB847674.

Figures 4, 5.

**Figure 4:**
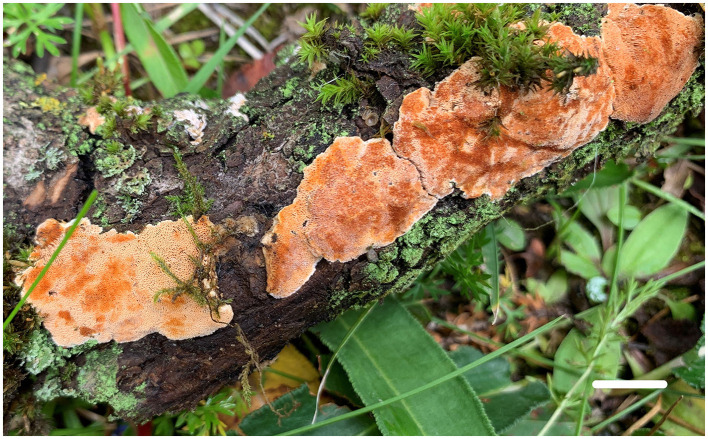
Basidiomata of *Steccherinum juniperi* (Paratype, Dai 23930) (Scale bar = 1.0 cm). Photographed by Yu-Cheng Dai.

**Figure 5:**
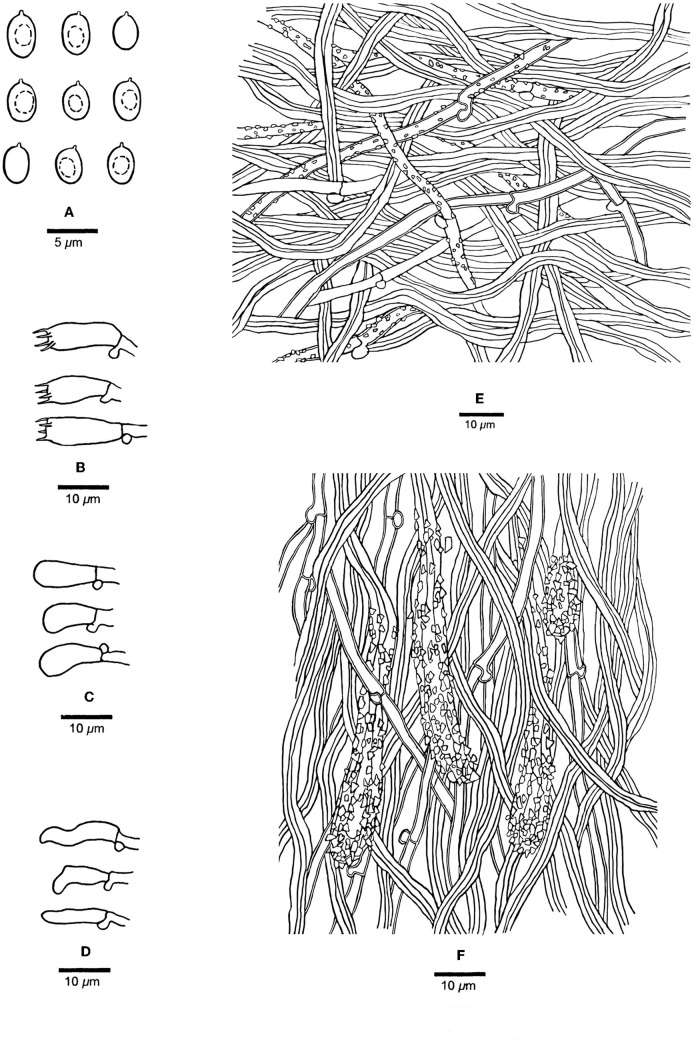
Microscopic structures of *Steccherinum juniperi* (Holotype, Dai 23931). **(A)** Basidiospores. **(B)** Basidia. **(C)** Basidioles. **(D)** Cystidioles. **(E)** Hyphae from subiculum. **(F)** Hyphae from trama. Drawn by Meng Zhou.

*Holotype*—China. Qinghai Province, Yushu, Leba Valley, on rotten wood of *Juniperus*, 5.VIII.2022, Dai 23931 (BJFC039175).

*Etymology—Juniperi* (Lat.): referring to the species growing on *Juniperus*.

*Fruiting body*—Basidiomata annual, resupinate, difficult to separate from the substrate, soft corky when fresh, hard corky when dry, up to 10 cm long, 2 cm wide, and ~2.5 mm thick at the center; pore surface buff yellow when fresh, apricot orange when bruised, buff to honey yellow upon drying; sterile margin distinct, cream and nearly 1 mm width; pores angular, 3–6 per mm; dissepiments thin, entire; subiculum very thin to almost absent, paler than tubes, nearly 0.5 mm thick; tubes concolorous with poroid surface, up to 2 mm long.

*Hyphal structure*—Hyphal system dimitic; generative hyphae with clamp connections; skeletal hyphae dominant, IKI–, CB+; tissues unchanged in KOH.

*Subiculum*—Generative hyphae hyaline, thin- to slightly thick-walled, rarely branched, sometimes encrusted with crystals, 2–3.5 μm in diam; skeletal hyphae dominant, hyaline, thick-walled with a medium to narrow lumen, unbranched, flexuous, interwoven, 3–5 μm in diam.

*Tubes*—Generative hyphae hyaline, slightly thick-walled, occasionally branched, 2–3 μm in diam; skeletal hyphae dominant, hyaline, thick-walled with a medium lumen, unbranched, flexuous, interwoven, 2–4.5 μm in diam. Skeletocystidia present in trama only, abundant, clavate to cylindrical, thick-walled with a wide lumen, strongly encrusted in the obtuse apex, 20–120 × 7–10 μm (encrusted part); cystidioles present, fusiform to slim clavate, hyaline, thin-walled, 10–15 × 3–4 μm; basidia clavate, hyaline, with a basal clamp connection and four sterigmata, 12–13 × 4–6 μm; basidioles dominant, similar to basidia in shape, but slightly smaller.

*Spores*—Basidiospores ellipsoid with an apiculus, hyaline, thin-walled, smooth, some with a medium guttule, IKI–, CB–, 3–4(−4.8) × (1.8–)2–3(−3.2) μm, L = 3.57 μm, W = 2.46 μm, Q = 1.38–1.52 (n = 60/2).

*Additional specimen (paratype) examined*—China. Qinghai Province, Yushu, Leba Valley, on rotten wood of *Juniperus*, 5.VIII.2022, Dai 23930 (BJFC039174).

***Steccherinum austrosinense*** (F. Wu, P. Du & X.M. Tian) Z.B. Liu, Y.C. Dai & Jing Si, comb. nov.

MycoBank: MB847672.

*Basionym—Junghuhnia austrosinensis* F. Wu, P. Du & X.M. Tian, MycoKeys 72: 5 (2020).

*Materials studied*—China. Yunnan Province, Jinghong, Virgin Forest Park, on fallen bamboo, 17.VI.2017, Dai 17540 (BJFC025072, holotype); Hainan Province, Wuzhishan County, Wuzhishan Forest Park, on fallen angiosperm branch, 9.IX.2019, Dai 17679 (BJFC025211, paratype).

***Steccherinum nandinae*** (F. Wu, P. Du & X.M. Tian) Z.B. Liu, Y.C. Dai & Jing Si, comb. nov.

MycoBank: MB847673.

*Basionym—Junghuhnia nandinae* F. Wu, P. Du & X.M. Tian, MycoKeys 72: 8 (2020).

*Materials studied*—China. Chongqing, Nanchuan County, Jinfoshan Forest Park, on dead tree of *Nandina domestica*, 1.XI.2019, Dai 21107 (BJFC032766, holotype), Dai 21108 (BJFC032767, paratype).

## 4. Discussion

In the present study, two new species (*S. juniperi* and *S. incrustans*) and two new combinations (*S. austrosinense* and *S. nandinae*) nested in the *Steccherinum* clade, based on the phylogenetic analysis of ITS + nLSU sequences data (Figure 1).

An ITS sequence JN710550 of the sample Miettinen 10301, named *Junghuhnia* cf. *nitida* from GenBank, is almost identical to Dai 19442 in the ITS regions, and the similarity between their sequences is up to 99.73%. Both samples were collected from Xishuangbanna, Yunnan Province, China. We believed that the sample Miettinen 10301 represented the same species as our specimen (Dai 19442), and they formed a lineage with strong supports (100% ML, 98% MP, and 1.00 BPP, Figure 1) in our phylogeny. Morphologically, *S. incrustans* can be distinguished from *Junghuhnia nitida* (Pers.) Ryvarden by having smaller pores (8–10 per mm vs. 5–7 per mm, Ryvarden and Johansen, 1980). In addition, *S. incrustans* differs from *J. nitida* by its tramal generative hyphae frequently covered with crystals, while they are smooth in *J. nitida*.

The phylogenetic analyses indicated that two specimens of *S. juniperi* formed a lineage with strong supports (100% ML, 100% MP, and 1.00 BPP) and grouped with *S. incrustans* with strong supports (100% ML, 100% MP, and 1.00 BPP) (Figure 1). *Steccherinum juniperi* differs from *S. incrustans* by its larger pores (3–6 per mm in *S. juniperi vs*. 8–10 per mm in *S. incrustans*). In addition, *S. juniperi* grows on gymnosperm in boreal forests, while *S. incrustans* grows on angiosperm in tropical forests.

*Steccherinum juniperi, S. austrosinense*, and *S. neonitidum* Westphalen & Tomšovský have poroid hymenophore and microscopically share cystidioles and similar sizes of basidiospores, but *S. austrosinense* and *S. neonitidum* have distinctly smaller pores (9–11 per mm in *S. austrosinense*, 8–10 per mm in *S. neonitidum*, vs. 3–6 per mm in *S. juniperi*). In addition, *S. austrosinense* and *S. neonitidum* grow on angiosperm, and their skeletocystidia are present in both tube trama and out of hymenium (Westphalen et al., 2018; Du et al., 2020), while *S. juniperi* grows on gymnosperm and only has skeletocystidia in tube trama.

*Steccherinum collabens* (Fr.) Vesterh. resembles *S. juniperi* in the field because they share similar pores when fresh and grow on gymnosperms; however, *S. collabens* has cylindrical to suballantoid basidiospores (3.2–3.6 × 1.4–1.7 μm, Niemelä, 2016).

The vegetation in Northwest China is relatively simple compared with the other parts of China, and a few limited new taxa of wood-habiting fungi were described from the area (Dai et al., 2007a,b, 2021), especially only a few species were recorded on *Juniperus* in China (Dai, 2012; Cui et al., 2019; Wu et al., 2022). *Steccherinum juniperi* is described as *Juniperus* in a dry environment of Northwest China, thus indicating that some special species adapted to the special host in the arid area.

## Data availability statement

The datasets presented in this study can be found in online repositories. The names of the repository/repositories and accession number(s) can be found below: https://www.ncbi.nlm.nih.gov/genbank/, ON182084, ON182087, OP956031, OP956076, and OP956077.

## Author contributions

Z-BL: investigation, software, data curation, visualization, and writing—original draft. Q-YZ and MZ: data curation and visualization. JS: visualization, supervision, writing—reviewing and editing, project administration, and funding acquisition. All authors contributed to the manuscript and approved the submitted version.
